# An Ultracompact Angular Displacement Sensor Based on the Talbot Effect of Optical Microgratings

**DOI:** 10.3390/s23031091

**Published:** 2023-01-17

**Authors:** Zhiyong Yang, Xiaochen Ma, Daguo Yu, Bin Cao, Qianqi Niu, Mengwei Li, Chenguang Xin

**Affiliations:** 1 School of Instrument and Electronics, North University of China, Taiyuan 030051, China; 2 School of Mechanical Engineering, North University of China, Taiyuan 030051, China; 3 School of Instrument and Intelligence, North University of China, Taiyuan 030051, China

**Keywords:** angular displacement sensor, Talbot effect, microgratings

## Abstract

Here, we report an ultracompact angular displacement sensor based on the Talbot effect of optical microgratings. Periodic Talbot interference patterns were obtained behind an upper optical grating. By putting another grating within the Talbot region, the total transmission of the two-grating structure was found to be approximatively in a linear relationship with the relative pitch angle between the two gratings, which was explained by a transversal shift of the Talbot interference patterns. The influence of the grating parameters (e.g., the grating period, the number of grating lines and the gap between the two gratings) was also studied in both a simulation and an experiment, showing a tunable sensitivity and range by simply changing the grating parameters. A sensitivity of 0.19 mV/arcsec was experimentally obtained, leading to a relative sensitivity of 0.27%/arcsec within a linear range of ±396 arcsec with the 2 μm-period optical gratings. Benefitting from tunable properties and an ultracompact structure, we believe that the proposed sensor shows great potential in applications such as aviation, navigation, robotics and manufacturing engineering.

## 1. Introduction

Real-time pitch angle measurements and orientation identifications are essential for applications such as the assembly of precision mechanical components, the position calibration of space probes and the positioning of machining platforms [1,2,3]. The recent interest in developing compact and energy-efficient mechatronic systems calls for an increasing interest in developing high-precision micro-angle sensors with ultracompact structures. The angle measurements generally include the measurement of the roll angle, yaw angle and pitch angle [4]. The high-performance measurement of the pitch angle remains a problem due to the limitation of the drifting of the laser beam [5,6]. In past years, several techniques, including photoelectric autocollimation [7], laser interferometers [8] and grating interferometers [9], have been reported for pitch angle measurements.

For photoelectric autocollimation [10], the collimated light beam is detected by a photodetector after being reflected by a plane mirror [11,12,13,14,15]. A change in the pitch angle leads to a rotation of the mirror, which results in a change in the reflection direction of the reflected beam. As a result, the location of the optical spot on the photodetector changes. The pitch angle is obtained from the position of the spot. For example, Yin et al. [16] combined the autocollimation method with the Moiré measurement technique. Using a right-angle prism, a pitch angle measurement with an error less than 5 arcsec within a 1000 arcsec range was demonstrated. In 2020, Guo et al. [17] replaced the plane mirror with a combined target reflector, improving the accuracy to 0.74 arcsec within a range of ±200 arcsec. Generally, autocollimation approaches offer a high precision. However, a relatively large size and complex setup is typically necessary by using reflected beams [18].

Another method to measure minor pitch angles is based on optical interference [19,20,21,22,23]. A laser interferometer was first reported for angle measurements within a relatively large range [24]. In 2010, Hahn et al. proposed a high-resolution angle measurement system based on heterodyne interferometer displacement sensors in which the change in angle was deduced by measuring the change in the displacement of four points on a single plane [25]. Subsequently, Hsieh et al. [26] demonstrated that a change in the displacement and angle could be simultaneously measured by using technology combining a heterodyne interferometer, a Michelson interferometer and a grating shearing interferometer [27]. These sensors generally have a large sensing range of up to 0.15 mrad. However, the application of these sensors is generally limited by a complex optical system, which typically requires multiple photodetectors and a careful optical alignment.

In 1999, Lin et al. found that the Moiré fringes observed behind a test grating were sensitive to small angles [28]. With the development of charge-coupled device (CCD) technology, the rolling angle was measured by using Moiré fringes combined with CCD technology [29]. Later in 2006, Wang et al. proposed a sensor that used the Talbot effect to detect the local light intensity and incident angle of a light source [30]. However, a discussion on the influence of structural parameters (e.g., the grating period, the number of grating lines and the distance between the two gratings) is absent.

To develop compact and energy-efficiency devices, pitch angle sensors with an ultracompact structure and a simple setup are in great demand [31,32]. In this paper, we propose an ultracompact pitch angular displacement sensor based on the Talbot effect of optical microgratings. The total transmission of a two-grating structure was found to be in a linear relationship with the relative pitch angle between the two gratings. With no need for optical components such as beam splitters, mirrors and half-wave plates, our design had an ultracompact structure with a common optical path. By using different parameters (such as the grating period, number of grating lines and distance between two gratings) of the gratings, a tunable sensitivity as well as a measuring range were demonstrated. A sensitivity of 0.19 mV/arcsec and a relative sensitivity of 0.27%/arcsec were obtained from the experimental results, indicating a potential for the proposed sensor in applications such as precision mechanical assembling and positioning associated with tunable properties. It also had an ultracompact structure.

## 2. Principle

**Figure 1:**
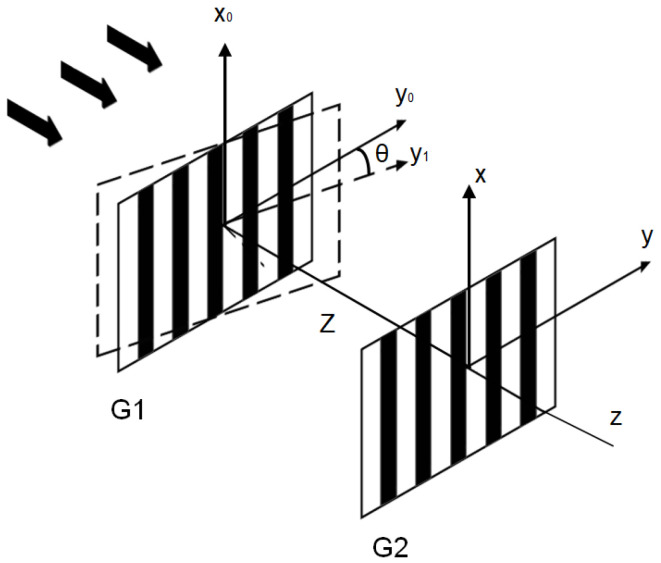
Schematic of the two-grating structure. G1 is the upper grating. G2 is the lower grating. G1 twisted around the x_0_ axis with a pitch angle of *θ*.

Figure 1 shows the schematic of the two-grating structure. A plane wave interference theory was used to analyze the two-grating structure. The amplitude transmission of the upper grating (G1) could be expressed as [33]

(1) $$t(x)=\sum_{n=-\infty }^{\infty }C_{n}\exp\left(i2\pi \frac{n}{d}x\right)$$

where *d* is the grating period and *C_n_* is the Fourier coefficient.

For the amplitude distribution in a plane at a certain distance (*z*) behind G1, the corresponding plane wave propagation factor should be multiplied by the plane wave function [34]. Under the condition of Fresnel diffraction, the propagation factor of a plane wave can be defined by $exp(ikz)exp(-i\pi \lambda zf^{2})$, where *f* is the spatial frequency along the x direction [35] and *λ* is the wavelength of the input laser beam.

Assuming that G1 was rotated along the x_0_ axis with an angle of *θ*, the two gratings were no longer parallel to each other. As *θ* was small, a simplified model was proposed to analyze the Talbot effect, in which G1 was projected into the x_0_-y_1_ plane after being rotated. In this case, the modified grating period (*d_1_*) was given by *d*cos*θ*. Therefore, the complex amplitude distribution at the plane could be given by

(2) $$U(x)=\exp(ikz)\sum_{n=-\infty }^{\infty }C_{n}\exp\left(-i\pi \lambda z\frac{n^{2}}{d_{1}^{2}}\right)\exp\left(i2\pi \frac{n}{d_{1}}x\right)$$

As shown in Equation (2), a periodical distribution of amplitude could be obtained behind the grating. The positions of the Talbot images (Talbot planes) could be defined by

(3) $$z_{j}=2j\frac{{\left(d\cos\theta \right)}^{2}}{\lambda }=2j\frac{d_{1}^{2}}{\lambda },j=0,1,2\ldots$$

As $\exp\left(-i\pi \lambda z\frac{n^{2}}{d_{1}^{2}}\right)=1$, the amplitude at the Talbot planes could be given by

(4) $$U(x)=\exp\left(ikz_{j}\right)\sum_{n=-\infty }^{\infty }C_{n}\exp\left(i2\pi \frac{n}{d\cos\theta }x\right)$$

As shown in Equations (1) and (4), the period of the amplitude distribution was identical to the period of G1 as *θ* = 0°. As *θ* ≠ 0°, the period of the amplitude distribution changed. At the same time, the Talbot planes twisted with a same angle of *θ*. As a result, a transversal shift of the Talbot images was induced.

**Figure 2:**
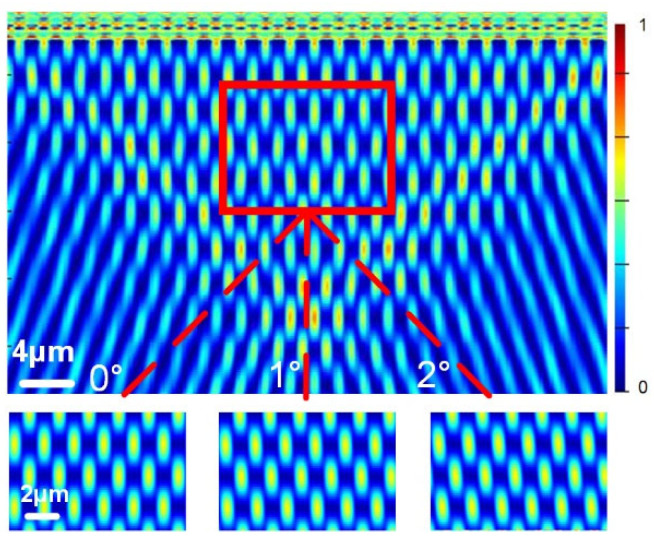
Simulated normalized intensity field of Talbot images behind G1. Simulations of Talbot images with *θ* of 0°, 1° and 2°, respectively.

Figure 2 shows the simulation results of the Talbot images behind G1 using the finite difference time domain (FDTD) method. Within a ratio from ~2 to ~3 of the wavelength of the input light to the grating period, Talbot images could be observed [36]. In the simulation, the substrate thickness was 1 μm, the Al grating thickness was 150 nm, the grating period was 2 μm, the number of grating lines was 10 and the incident wavelength was 1550 nm. The Talbot images were located within a triangle area. As the grating was rotated by 0°, 1° and 2° along the x_0_ axis, the Talbot images rotated at the same angle, respectively.

**Figure 3:**
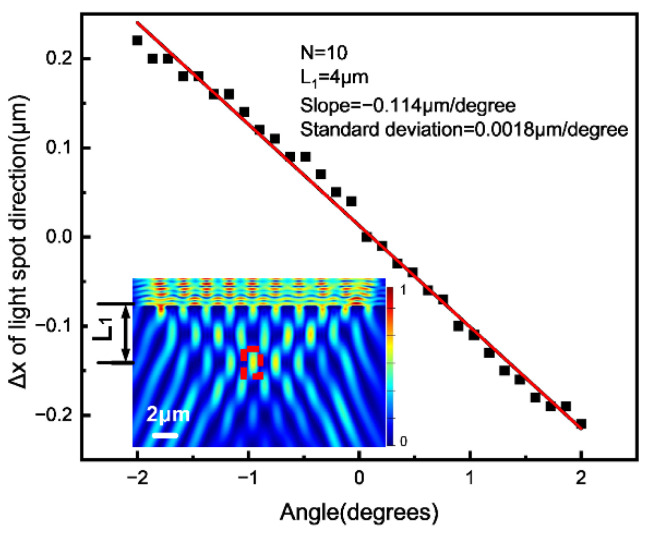
The relationship between the pitch angle and the shift of the Talbot image. The black dots indicate the simulated value of the transversal shift of the Talbot image. The red line indicates the linear fitting straight line (R^2^ > 99%). In the simulation, the number of grating lines was 10 and the distance from the center of the Talbot image to G1 was L_1_ = 4 μm.

The relationship between the transversal shift of the Talbot images and the pitch angle was investigated in a simulation. As shown in Figure 3, the center of one Talbot image, which is indicated by the red dotted line inside the figure, shifted linearly along the x direction as the pitch angle changed from −2° to 2°. A slope of −0.114 μm/degree and a standard deviation of 0.0018 μm/degree were obtained.

The grating period of the lower grating (G2) was same as the period of G1. Assuming that the distance between G1 and G2 was *Z*, the complex amplitude distribution of the light field behind G2 could be expressed as [37]

(5) $$U^{\prime }(x)=\exp(ikZ)\sum_{n-\infty }^{\infty }\sum_{m=-\infty }^{\infty }C_{n}C_{m}\exp\left(i2\pi \frac{(n/\cos\theta )+m}{d}x\right)$$

The total transmitted light intensity (*I*) was given by

(6) $$I(x)=\sum_{n=-\infty }^{\infty }\sum_{m=-\infty }^{\infty }\sum_{p=-\infty }^{\infty }\sum_{q=-\infty }^{\infty }C_{n}C_{m}C_{p}C_{q}\exp\left[i2\pi \frac{(m-q)+(n-p)/\cos\theta }{d}x\right]$$

where *m, n, p* and *q* are the periods of the Fourier series.

**Figure 4:**
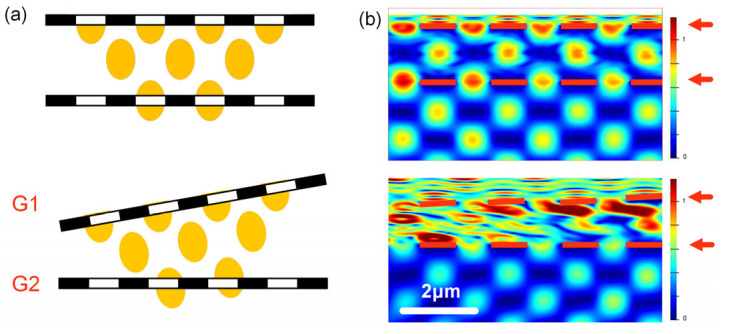
(**a**) Schematic diagrams of the simulated figures with *θ* = 0° and *θ* = 2°, respectively. (**b**) Transmission light of a two-grating structure consisting of two optical microgratings with a different *θ* of G1. The positions of the two gratings are indicated by the red arrows.

As shown in Figure 4, transmissions of the two-grating structure with different pitch angles were obtained. In the case where *θ* = 0°, most of transmitted light from G1 went through G2, leading to a relatively high total transmission. In contrast, as *θ* = 2°, the transmitted light from G1 was blocked by G2, resulting in a significant decrease in the total transmission.

## 3. Simulation Results

The effect of grating parameters such as the distance between the two gratings, the period of the gratings and the number of grating lines on the total transmission was investigated by using the FDTD method.

### 3.1. Effect of the Distance between the Two Gratings

**Figure 5:**
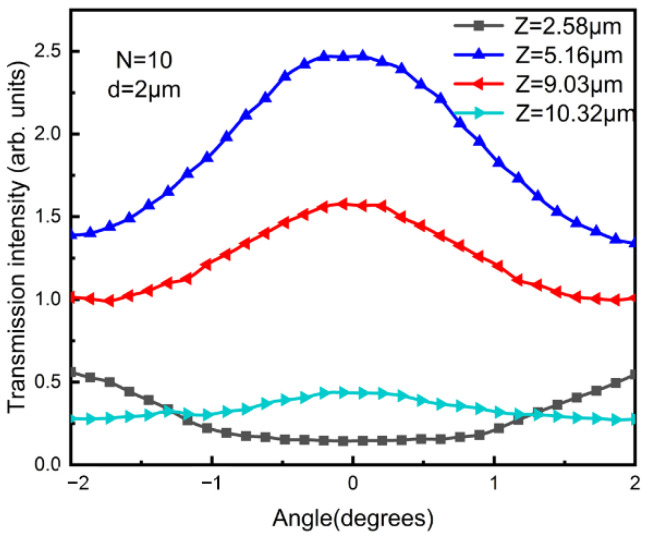
Simulated transmission intensity with different distances between the two gratings in which *Z* = 2.58 µm, 5.16 µm, 9.03 µm and 10.32 µm, respectively.

The simulated transmission intensity with different distances between the two gratings is shown in Figure 5. In the simulation, the grating period was 2 μm, the number of grating lines was 10 and the wavelength was 1550 nm. With a different distance, the response of the transmission intensity to the input pitch angle changed as well. For example, as *Z* changed from 5.16 μm to 10.32 μm, the maximum intensity changed from 2.5 to 0.55. A maximum contrast (defined as the difference between the maximum value and the minimum value in one single curve) was obtained as 1.1 with *Z* = 5.16 μm. We found that a pitch angle of 0° was always an extreme point. When G2 was located at the Talbot positions (*Z* = M*Z_T_*; M = 0, 1, 2…; *Z_T_* was the period of the Talbot images along the z direction), the transmission intensity decreased as the pitch angle deviated away from 0° (e.g., *Z* = 5.16 μm). In contrast, the transmission intensity increased with an increasing pitch angle when G2 was located at the semi-Talbot positions of G1 (*Z* = (M + 1/2) *Z_T_*; M = 0,1,2…). In order to obtain a better sensitivity, a curve with a larger contrast was preferred.

### 3.2. Effect of the Number of Grating Lines

**Figure 6:**
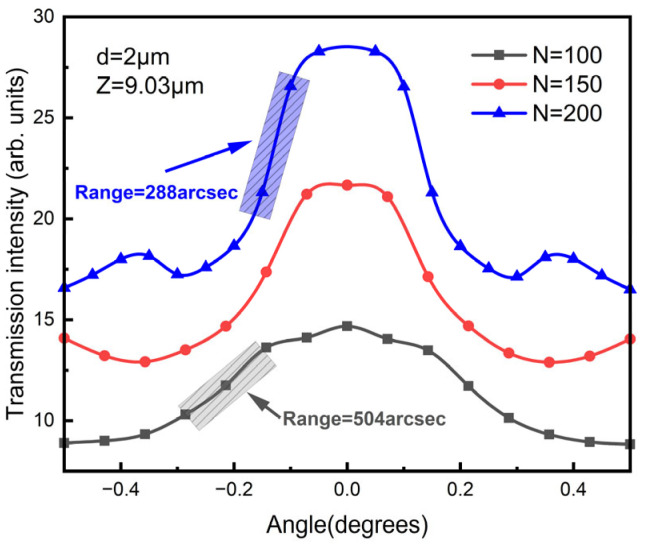
Simulated transmission intensity with different numbers of grating lines in which *N* was 100, 150 and 200, respectively.

Figure 6 shows the simulated results of transmission intensity with different numbers of grating lines (*N*). The distance *Z* was set to be 9.03 µm to guarantee an input angle up to ±0.5° as well as a relatively high sensitivity. The simulated results showed that the relative sensitivity changed from 0.23%/arcsec to 0.34%/arcsec as the number of grating lines increased from 100 to 200. At the same time, the range (defined as the linear fitting within which R^2^ > 99%) of the angle measurement decreased from 504 arcsec to 288 arcsec. As the number of grating lines increased, the relative sensitivity increased with a decreased detection range.

### 3.3. Effect of the Grating Period

**Figure 7:**
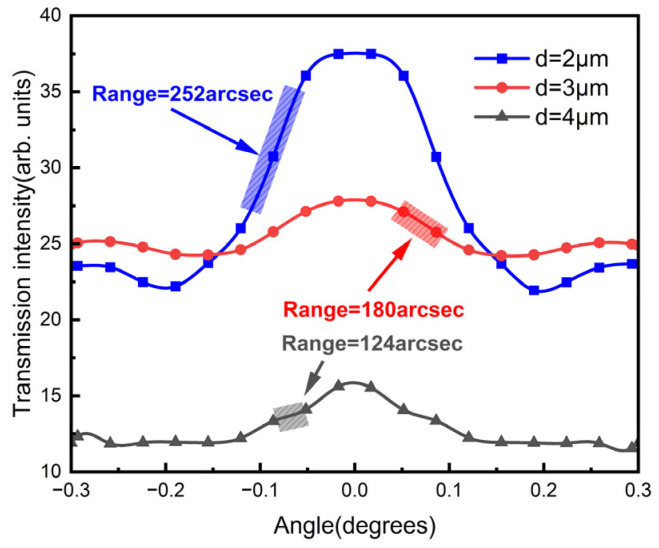
Simulated transmission intensity with different grating periods in which *d* = 2 µm, 3 µm and 4 µm, respectively.

As shown in Figure 7, the relationship of the transmission intensity to the pitch angle changed when using different grating periods. This was explained by a changing *Z_T_* with a different *d*. To obtain a higher sensitivity, *Z* was set to be a different distance as *d* = 2 μm, 3 μm and 4 μm, correspondingly, using the same grating area with total length of 0.5mm. The wavelength of the input laser was set to be 1550 nm. The contrast in the 2 μm-period case was 16 and 4.6 times larger than those in the 3 μm and 4 μm cases, respectively. In addition, the range changed from 252 arcsec to 180 arcsec and 124 arcsec as the grating period went from 2 μm to 3 μm and 4 μm, respectively. The results showed that with a smaller grating period, there was an increased sensitivity and detection range.

## 4. Experimental Results

**Figure 8:**
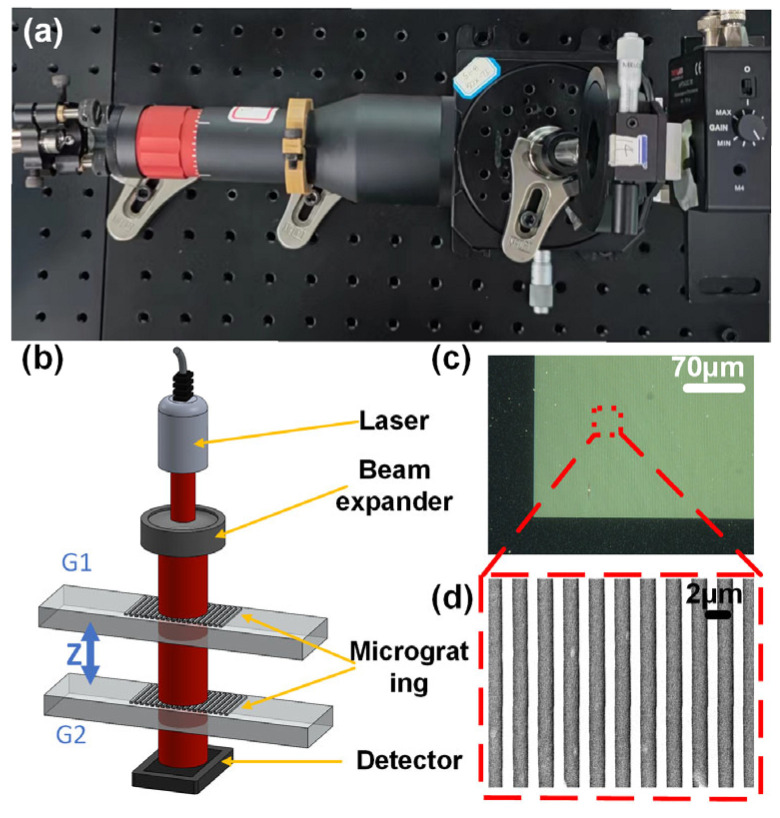
(**a**) Optical image of the experimental setup. (**b**) Schematic diagram of the proposed sensor. (**c**) Image of the micrograting used in the experiment from an optical microscope. (**d**) Image of the micrograting from a scanning electron microscope.

The schematic diagram of the experimental system is shown in Figure 8. The beam from a 1550 nm wavelength laser (LR-SFJ-1550, Leishiguang, Changchun, JL, China) was incident on the upper grating after passing through a beam expander. A periodic light intensity distribution was formed behind the upper grating based on the Talbot effect. The lower grating was placed within this Talbot region. The distance between the two gratings required a careful setup for a better sensitivity. When the pitch angle of the upper grating changed, the transmission behind the lower grating changed as well, which was detected by a photodetector (APD430C/M, Thorlabs, Newton, NJ, USA).

The experimental setup is shown in Figure 8a,b. The microgratings (shown in Figure 8c,d) used in the experiment were amplitude gratings made from Al, which were prepared by a lithography process. The thickness of the Al film and the SiO_2_ substrate was 150 nm and 500 μm, respectively. Gratings with periods of both 2 μm and 3 μm were used in the experiment.

**Figure 9:**
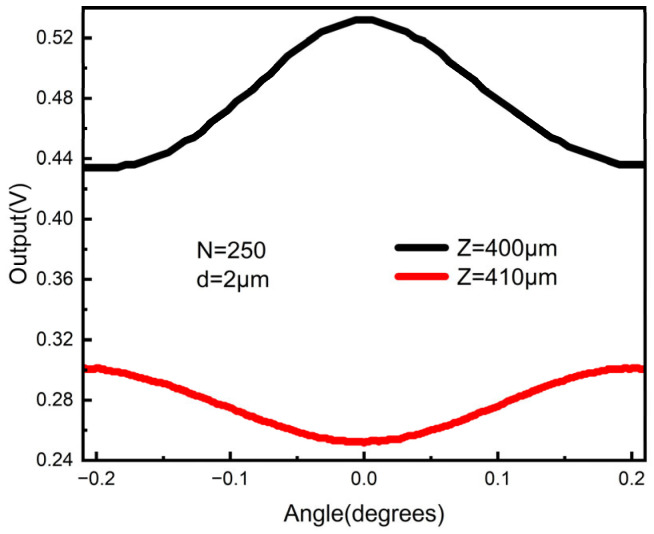
The output of the photodetector at *Z* = 400 μm and 410 μm, respectively.

The influence of *Z* on the output of the photodetector was investigated in an experiment. Figure 9 shows the relationship of the transmitted intensity with the pitch angle at *Z* = 400 µm (~95 *Z_T_*) and 410 µm (~97.5 *Z_T_*), respectively. The output tended to decrease and increase as the pitch angle deviated away from 0° in the two cases, which was in agreement with the simulated results. In addition, the contrast in the 400 μm case was 1.67 times higher than that in the 410 μm case. However, the range for the former was only 0.86 times greater than that of the latter. The results showed that a tunable sensitivity and range could be obtained by changing the distance between the two gratings.

**Figure 10:**
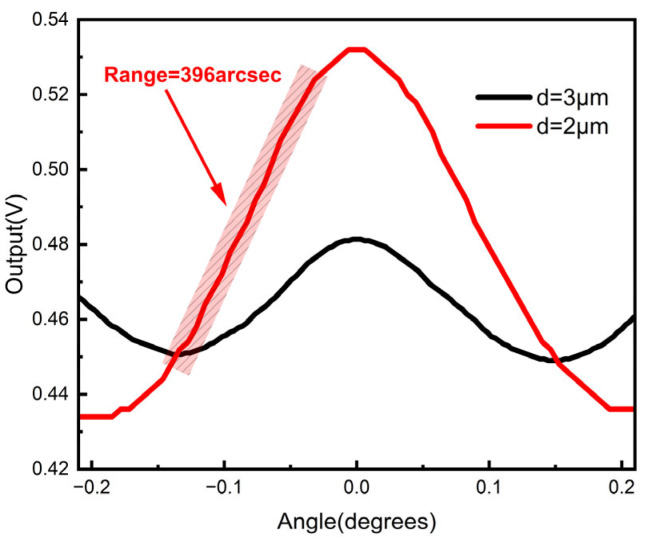
The output of the photodetector with *d* = 2 μm and 3 μm.

Figure 10 shows the experimental results with grating periods of 2 μm and 3 μm, respectively. *Z* was set to be 400 μm and 1020 μm, correspondingly. In the two cases, the lower grating was always located at the Talbot positions, which was in agreement with the simulation. The linear range in the 2 µm-period case was found to be 396 arcsec, which was 1.5 times greater than that in the 3 µm-period case. The results showed a good agreement with the simulated results, as shown in Figure 7.

**Figure 11:**
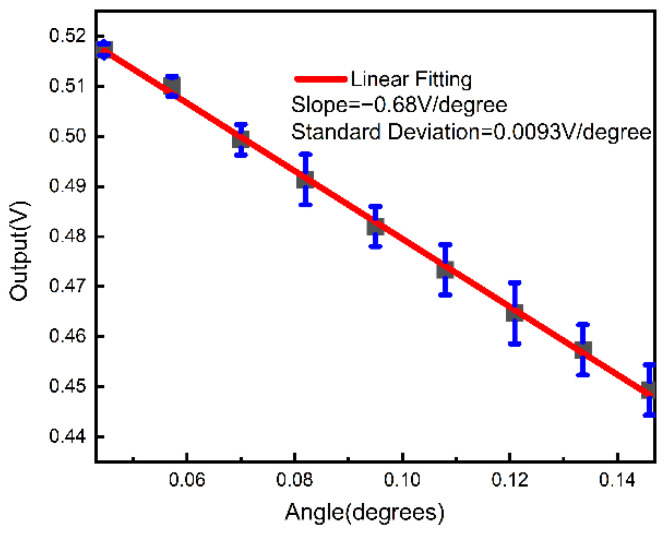
Linear fitting between transmitted intensity and the input pitch angle within a range from 0.04° to 0.15°. The experiment was repeated three times. The black dots show the average value of experimental results.

In the 2 μm-period case, a linear range was obtained within a range from 0.04° to 0.15°, as shown in Figure 11. An error within ±6 mV was obtained in the experiment. A slope of 0.68 V/degree and a standard deviation of 0.0093 V/degree were obtained by the linear fitting of the average experimental values. The sensitivity (*k_0_*) in an absolute unit (mV/arcsec) of the linear region was calculated to be about |−0.68 V/degree| = 0.68 V/3600 arcsec = 0.19 mV/arcsec. The relative sensitivity (*k_1_*) could be given by

(7) $$k_{1}=\frac{k_{0}}{V_{0}}\times 100\%$$

where *V_0_* is the maximum voltage difference across the whole linear region; *V_0_* was measured to be 71 mV. As a result, *k_1_* was calculated to be 0.27%/arcsec within a linear range of 396 arcsec, which was comparable with those from the autocollimation approaches [17].

## 5. Discussion

The difference between the *k_0_* may have resulted from the different sensitivities of the photodetector used. In the experiment, a detector with a noise equivalent power of 8.1 pW/√Hz was used. As a result, the relative sensitivity was calculated to be 0.27%/arcsec. By using a detector with a better responsivity, a better sensitivity could be obtained in principle.

Table 1 shows a comparison between the proposed sensor and several typical miniaturized optical angular sensors. These fiber-optic sensors generally have a large sensing range of up to ±7.2° [38,39]. The proposed sensor showed a much better sensitivity and a smaller range than the fiber-based type and was comparable with an autocollimator [17]. The results indicated its use for potential applications requiring a high sensitivity within a small detection range such as fine adjustments for the ultraprecision positioning of work tables in lithography machines or scanning probe microscopes [40,41].

## 6. Conclusions

Based on the Talbot effect of microgratings, a pitch angular displacement sensor was demonstrated. Using a two-grating structure, the total transmission was found to be in an approximately linear relationship to the relative pitch angle between the two gratings. The influence of different grating parameters (e.g., the grating period, the number of grating lines and the distance between the two gratings) was analyzed from both a simulation and an experiment. The total sensitivity was experimentally measured to be 0.19 mV/arcsec. A relative sensitivity of 0.27%/arcsec was obtained within a linear range of ±396 arcsec using 2 μm-period microgratings. It is worth mentioning that a better sensitivity and detection range could be obtained in principle by using optical gratings with a smaller period. Compared with traditional techniques based on autocollimation or laser interference, the proposed sensor showed a much more compact structure with a comparable sensitivity and detection range, indicating a great potential for the sensor in applications such as precision mechanical assembly and positioning.

## Figures and Tables

**Table 1:** Comparison of miniaturized optical sensors.

Reference	Year	Relative Sensitivity or Sensitivity	Type
[38]	2016	13.71%/deg	Fiber-based
[17]	2020	0.714 mV/arcsec	Autocollimator
[39]	2021	0.08104 V/degree	Fiber-based
This work	2023	0.27%/arcsec 0.19 mV/arcsec	Grating-based

## Data Availability

Not applicable.
